# Managing multidrug resistant Gram-negative bacteraemia in immunocompromised hosts

**DOI:** 10.1097/QCO.0000000000001205

**Published:** 2026-05-07

**Authors:** Cecilia Bonazzetti, Alice Toschi, Maddalena Giannella

**Affiliations:** a Infectious Diseases Unit, IRCCS Azienda Ospedaliero-Universitaria di Bologna; b Department of Medical and Surgical Sciences, University of Bologna, Bologna, Italy

**Keywords:** empirical antibiotic therapy, febrile neutropenia, immunocompromised patients, multidrug-resistant Gram-negative bacteria, solid organ transplantation

## Abstract

**Purpose of review:**

Multidrug-resistant Gram-negative bloodstream infections (MDR-GNBSI) are increasingly frequent in immunocompromised hosts, particularly solid organ transplant (SOT) recipients and patients with haematological malignancy (HM) receiving intensive chemotherapy and/or cellular therapies. We summarize current evidence and practical approaches across empirical, quasi-targeted and targeted management.

**Recent findings:**

Contemporary cohorts show rising rates of MDR-GNBSI in both SOT and HM patients. Appropriate therapeutic management is key to ensure good clinical outcome while minimizing ecological impact. Risk-adapted empirical therapy usually incorporates prior colonization and local epidemiology plus individual risk factors, thus screening could play central role. Rapid diagnostic testing can reduce time to appropriate and proportionate therapy, but currently the majority of them provide genotypic resistance that in Gram negatives can be difficult to interpret, in addition off-target resistance mechanisms should be considered mainly in nonfermenter bacteria. Thus, machine-learning models and structured BSI bundles are emerging tools to support early decision-making and stewardship. For targeted therapy, novel β-lactam/β-lactamase inhibitor combinations and cefiderocol expand options for carbapenem-resistant strains; pharmacokinetics/pharmacodynamics (PK/PD) optimization may improve target attainment and mitigate resistance selection. Evidence supporting oral step-down and shorter courses is growing, whereas routine follow-up blood cultures remain controversial due to observational bias.

**Summary:**

MDR-GNBSI management in immunocompromised patients requires individualized, data-driven strategies integrating risk stratification, rapid microbiology, and PK/PD optimization. Pursuing antimicrobial stewardship principles in these populations remains a priority.

## INTRODUCTION

Gram-negative bacteria have emerged as main causes of bloodstream infections in several types of immunocompromised hosts (ICHs), particularly among solid organ transplant (SOT) recipients and in patients with haematological malignancy (HM) undergoing intensive chemotherapy and/or cellular therapies [1,2]. In these settings, the emergence and spread of multidrug resistance have increased in the past decades, being associated with high rates of morbidity and mortality [3,4^▪▪^,^5^].

ICHs are particularly vulnerable due to healthcare contacts, invasive procedures, and antibiotic exposures. Indeed, the frequency of multidrug-resistant Gram-negative bloodstream infections (MDR-GNBSI) is higher in ICHs when compared with non-ICHs and is usually associated with higher mortality rates [6]. Higher mortality rates were attributed to an increased probability of receiving initial inappropriate therapy in MDR-GNBSI compared to susceptible GN-BSI episodes in HM with severe neutropenia [7,8]. While for SOT recipients, results from heterogeneous cohorts of patients with MDR-GN infections, in particular carbapenem resistant Enterobacterales (CRE), suggest that mortality is lower in SOT than in non-SOT recipients [9]. However, a number of biases could influence these data: SOT recipients are younger and less comorbid than the general population, their infectious risk is better recognized and managed compared with other patient groups. Indeed, several studies showed that SOT recipients colonized and/or infected with MDR-GN have a significant higher risk of mortality compared with patients without peri-transplant MDR-GN isolation [10,11].

MDR-GNBSI usually emerges in, or is associated with, significant gut microbiome alteration [12]. In ICHs, it is currently evident that gut microbiome alteration represents a critical concern.

Indeed, in immunocompromised hosts it has been associated not only with the emergence of further antibiotic resistance, *Clostridioides difficile*, and fungal infections, as observed in other settings, but also with a negative impact on the underlying condition, including dysbiosis and impaired graft function [13]. In addition, prior exposure to antianaerobic agents (such as piperacillin/tazobactam or carbapenems) has been linked to the development of graft-vs.-host disease (GVHD) after allogeneic hematopoietic stem cell transplantation (allo-HSCT), as well as to severe cytokine release syndrome or immune effector cell–associated neurotoxicity syndrome in patients treated with CAR-T therapy [14–16].

In this framework, the management of MDR-GNBSI in ICHs is extremely challenging as favourable clinical outcome is often associated with a prompt initiation of active broad-spectrum treatment but at the same time minimize antibiotic overuse is desirable. Our aim is to discuss the management of MDR-GNBSI in the main ICH settings, such as SOT recipients and patients with HM, addressing the critical steps of empirical therapy choice (prelaboratory results), quasi-targeted therapy (preliminary, usually molecular-based rapid diagnostic testing results) and targeted therapy, as well as pharmacokinetics/pharmacodynamics (PK/PD) issues mainly regarding the use of novel drugs, shift to oral therapy, optimal treatment duration and follow-up blood cultures (FUBCs). 

**Box 1:**
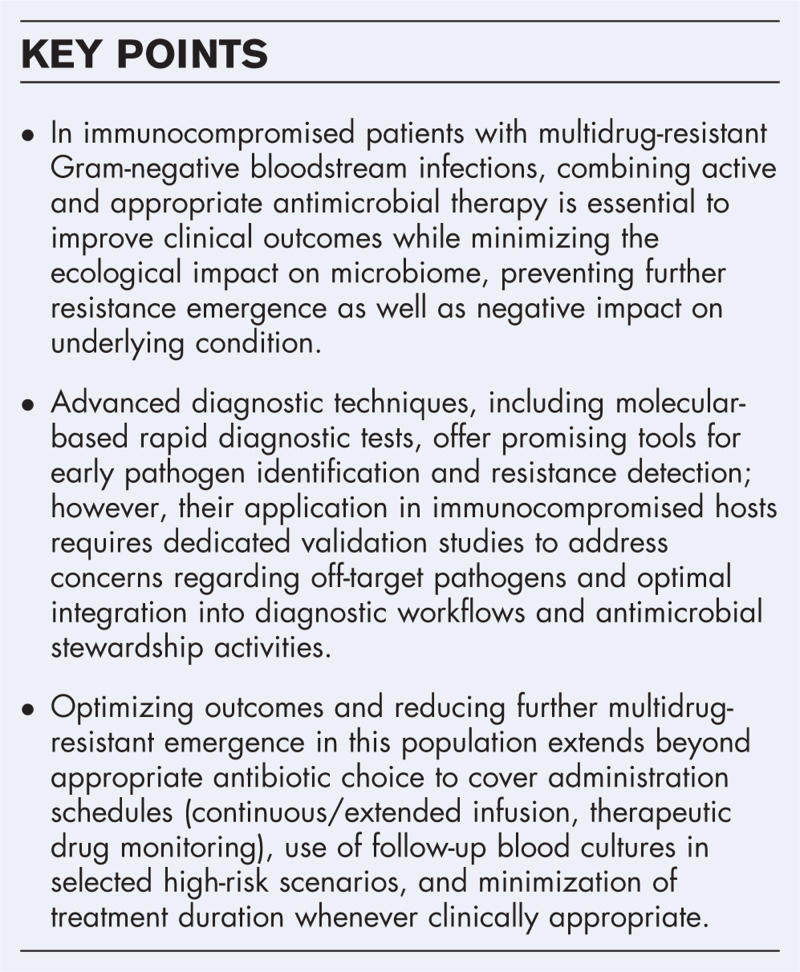
no caption available

## METHODS

We conducted a narrative literature review, including only English written papers, focusing on the most relevant and significant contributions published within the period 2015–2026. Multidrug resistance (MDR) was defined as acquired nonsusceptibility to at least one agent in three or more antimicrobial categories, according to Magiorakos classification [17]. Difficult to treat resistance was defined as a treatment-limiting resistance to all first-line agents, including all beta-lactams and fluoroquinolones [18].

## MDR-GNBSI BURDEN IN MAIN IMMUNOCOMPROMISED HOSTS

### Solid organ transplant recipients

Bloodstream infections remain a frequent complication following solid organ transplantation, with Gram-negative bacteria accounting for the majority of episodes (Table 1) [3,9,19–27,28^▪^,^29^–^35^]. The overall incidence of Gram-negative BSI ranges from 6% to 20% within the first-year posttransplant, with liver transplant recipients exhibiting the highest burden compared to kidney recipients (12.3% vs. 2.6%). The median time from transplantation to BSI onset is approximately 26–39 days, with early-onset infections (≤30 days) occurring in 43–45% of cases. Regarding aetiology, *Escherichia coli* (29–64%) and *Klebsiella pneumoniae* (10–42%) represent the most frequently isolated pathogens, followed by *Pseudomonas aeruginosa* (10–34%) and *Acinetobacter baumannii* (8–20%) [36]. The burden of multidrug resistance has increased substantially over time, with MDR Gram-negative bacteria now accounting for 19–40% of all Gram-negative BSI episodes [31,37,38,39^▪^] (Table 1). Extended spectrum cephalosporin resistance Enterobacterales (ESCR-E) represent the most common resistance phenotype (15–45% of Enterobacterales BSI) [22,31,35,40], while carbapenem-resistant Enterobacterales (CRE) account for 8–22% of episodes [41], with higher rates observed in liver transplant recipients [25,28^▪^,^41^,^42^]. Notably, despite the high prevalence of MDR pathogens, overall 30-day mortality for Gram-negative BSI in SOT recipients remains relatively low, ranging from 10% to 26%. This finding likely reflects the characteristics of this population, which typically comprises younger patients with fewer comorbidities and benefits from close medical surveillance and rapid access to specialized care. However, therapeutic failure and infection recurrence represent significant concerns, with relapse rates reaching 8–13% at 30 days even in appropriately treated ESCR-E BSI of urinary origin [22]. Mortality increases substantially in the presence of carbapenem resistance, with CRE BSI associated with 30-day mortality rates of 33–66% and representing an independent predictor of death (adjusted hazard ratio 2.85; 95% CI 1.68–4.84) [22].

**Table 1:** Epidemiology and aetiology of MDRGNBSI in SOT patients

Ref	First author	Country	Journal and year of publication	Study type	Epidemiology	Aetiology
[19]	Bingbing Qiao *et al.*	China	BMC Infectious Disease; 2017	Retrospective, double-centre observational cohort; 2169 transplants over the study period.	Overall BSI burden: 327 BSI episodes in 227/2169 (10.5%) recipients. MDR GN bacteraemia burden: 99 episodes in 91/2169 (4.6%). 30-day (infection-related) mortality after first MDR GNB episode: 39.6% (36/91). Timing: median 26 days from transplant to MDR GNB diagnosis; liver median 11 days vs. kidney median 69 days (first episode). By transplant type (risk): liver recipients 44/357 (12.3%) vs. kidney recipients 47/1812 (2.6%) affected. Mortality predictors: septic shock, creatinine > 1.5 mg/dl, nosocomial origin, concomitant BSI.	Among 99 MDR GN isolates: *E. coli* 29 (29.3%), *A. baumannii* 24 (24.2%), *E. cloacae* 11 (11.1%), *K. pneumoniae* 10 (10.1%). Resistance: ESBL(+) rods: 80.2%; Carbapenem-resistant rods: 41.8%; Nonfermenters: 39.6%
[20]	Tsikala-Vafea *et al.*	Greece	TID; 2020	Single-centre retrospective observational cohort	2008–2018: 1962 kidney transplant recipients (KT); 195 Gram-negative BSI episodes in 182 patients; incidence 1.393/100 patient-years. Urinary source 70.9%; nosocomial 28%. MDR 19.2% of BSIs; risk factors for MDR included prior antibiotics and prior ICU stay. In-hospital mortality 6%.	Main sources: UTI 70.9%. Main pathogens: *E. coli* 63.7%, *K. pneumoniae* 20.3%, *P. aeruginosa* 8.2%. MDR proportion: 19.2% (35/182). Carbapenemase types among carbapenem-resistant isolates (n=13): VIM, NDM-1, KPC (some co-producing VIM-1).
[21]	Anesi, Judith A. *et al.*	USA	CID; 2021	Multicentre case–control study	988 EB BSI episodes among 897 SOT recipients, with 395/988 (40%) ESBL-EB. No overall transplant-program incidence of ESBL-GNBSI (denominator is EB BSI episodes, not all SOT), and no mortality/outcome endpoints are provided (it is risk-factor focused).	Overall: *Klebsiella* spp. 42%, *E. coli* 37%, Enterobacter spp. 13% In ESBL: *Klebsiella* spp. 46%; in non-ESBL: *E. coli* 41%. Resistance characteristics: FQ-R 26%, PTZ-R 17%, carba-R 8%, TMP-SMX resistance 52%.
[22]	Pierriotti L *et al.*	Multicentre international	TID; 2021	Retrospective international multicentre observational cohort	306 kidney transplant recipients with monomicrobial ESBL BSI secondary to UTI. Timing: median 119 days from transplant to BSI; 23.2% within the first posttransplant month. Therapeutic failure: 8.2% at day 7; 13.4% at day 30. Mortality: 1.0% at day 7; 2.9% at day 30 (most deaths attributed to ESBL-E BSI).	ESBL-E organisms: *E. coli* 62.1%, *Klebsiella* spp. 35.0%, others rare (Enterobacter spp. 1.3%).
[23]	Wang R. *et al.*	USA	TID; 2021	Multicentre case-control study (SOT recipients with Enterobacterales BSI 2005–2018; cases = ESBL-EB BSI; controls = non-ESBL EB BSI)	897 SOT recipients with EB BSI; 356 (40%) ESBL-producing. Derivation cohort: 539 (135, 25% ESBL-EB). Validation cohort: 358 (221, 62% ESBL-EB). Main isolates included *E. coli* and *Klebsiella* spp.	ESBL-producing Enterobacterales as BSI aetiology. Derivation cohort organisms: *E. coli* 42%, *Klebsiella* spp. 41%, Enterobacter spp. 9%, etc.; ESBL in 20% of *E. coli* and 28% of *Klebsiella* isolates. Validation cohort: *Klebsiella* spp. 42%, *E. coli* 32%, Enterobacter spp. 19%; ESBL in 64% of *Klebsiella* and 65% of *E. coli* isolates
[24]	Pagani Nicole *et al.*	Italy	Microorganism; 2021	Single-centre retrospective observational cohort of SOT recipients	CP-Kp enteric colonization: 45/828 (5.4%); mostly posttransplant (88.9%), median 12 days after transplant. CP-Kp invasive infection: 35/828 (4.2%); mortality among invasive infections 51.4%. CP-Kp BSI: 18/828 (2.2%); median onset 12 days posttransplant; BSI mortality 66.7% (30-day 33.4%, 1-year 61.1%).	Resistance profile reported for CP-Kp strains in BSI subset: colistin-resistant 27%; tigecycline resistant 11% / intermediate 33%; gentamicin intermediate 22% (no gentamicin resistance reported).
[3]	Oriol I *et al.*	Spain	Transplant International; 2017	Single-centre prospective observational cohort	Transplants performed (2007–2016): 1829 SOT (1113 kidney, 547 liver, 169 heart). BSI episodes in first year: 218 episodes (in 178 patients). Timing: median days from transplant to BSI onset 39 (IQR 12–133); increases over periods. 30-day mortality (overall): 22/218 (10.1%); 7-day mortality 5.0%; both stable over time. Septic shock at presentation: 17.8% overall	GNBSI increased over time from 54.1% → 93.3% *Pseudomonas aeruginosa*: 2.4% → 20.4% *Klebsiella pneumoniae*: 7.1% → 26.5% *E. coli*: up to 26.5% in the last period. MDR GNB increased from 4.8% → 38.8%.
[25]	Yanik Yalçin T. *et al.*	Turkey	Turkish Journal of Medical Sciences; 2021	Multicentre retrospective study (11 centres), adult SOT recipients with XDR GNB bacteraemia	164 SOT recipients; 171 bacteraemia episodes. Transplants: liver 56.7%, kidney 28%, heart 8.5%, lung 6.7%. Episodes in first year post-Tx 63.7%; early-onset ≤30d: 45%. Sources: surgical site 39.2%, urinary 23.4%, catheter-related 15.2%, respiratory 11.1%, intraabdominal 8.8%. Mortality: 7-day 18.1% (31/171); 30-day 26.3% (45/171).	XDR GNB distribution: *K. pneumoniae* 40.4%, *A. baumannii* 34.5%, *E. coli* 11.7%, *P. aeruginosa* 10.5%, Enterobacter spp. 2.9%; polymicrobial 8.2%.
[26]	Wang Q. *et al.*	China	Front Cell Infect Microbiol. 2023	Single-centre retrospective study (Jun 2019–Dec 2021)	40 KT treated with tigecycline combination therapy; CRGNB often donor-related: 28/40 (70%) isolates from organ preservation solution, with confirmed/possible transmission reported; infection sites included bloodstream, urinary tract, sputum/respiratory, wound; septic shock 30%; clinical response 80% (32/40); 28-day mortality 7.5% (3/40)	Carbapenem-resistant GNB (resistant to meropenem or imipenem): *K. pneumoniae* 75% (30/40), *A. baumannii* 15% (6/40), *E. coli* 10% (4/40); all isolates reported susceptible to tigecycline
[27]	Herrera S. *et al.*	Spain	Antibiotics; 2023	Retrospective analysis of a prospectively database: 1991–2019	2057 episodes of *P. aeruginosa* BSI; 265 (13%) in SOT recipients (kidney 130, liver 105, heart 9, kidney–pancreas 21). Overall 30-day mortality 20%; in SOT 13% vs. non-SOT 21%. Carbapenem-resistant (CR) episodes 475 (24%), with increasing trend and >30% CR in the last period.	N/A
[28^▪^]	Anesi J. A. *et al.*	USA	Transplantation; 2023	Multicentre retrospective cohort (CRE BSI vs. non-CRE Enterobacterales BSI)	Burden within Enterobacterales BSI in SOT: 897 Enterobacterales BSI episodes in SOT recipients; 70 (8%) were CRE BSI. 60-day mortality: 33/70 (47%) in CRE BSI vs. 102 (12%) in non-CRE Enterobacterales BSI. Adjusted association: CRE BSI associated with significantly increased hazard of death within 60 days (aHR 2.85, 95% CI 1.68–4.84).	N/A
[29]	Hu J. *et al.*	Chine	International Journal of Antimicrobial Agents; 2024	Retrospective single-centre cohort comparing CAZ-AVI vs. other active regimens (Jan 2018–Jun 2021)	SOT cohort size: 50 SOT recipients with CRKP infection (30 CAZ-AVI; 20 other regimens). Infection sites in SOT BSI 52%, respiratory 52%, IAI 44%, UTI 2%; 52% multisite infection. 30-day mortality: 38% overall; 23.3% with CAZ-AVI vs. 60% with other regimens (a OR 0.19, P=0.014). 90-day mortality: 35.7% CAZ-AVI vs. 86.7% other regimens (aOR 0.06, *P* = 0.003). Clinical cure: 90% CAZ-AVI vs. 40% other regimens	Focus pathogen is CRKP (carbapenem-resistant *K. pneumoniae*). Reports polymicrobial infections in SOT (32%) and lists co-pathogens (e.g., *P. aeruginosa*, *Acinetobacter* spp., *Enterococcus* spp., fungi, etc.).
[30]	Ayaz C. M. *et al.*	Turkey	BMC Infectious Disease; 2024	Retrospective, cross-sectional observational study	Population screened: 1104 SOT recipients (mainly kidney and liver). GN-BSI burden: 118 GN-BSI episodes in 113 patients. Timing: median 39 days posttransplant to GN-BSI overall; among hospitalized patients median 11 days. Early-onset GN-BSI (≤30 days): 49/113 (43.4%). Mortality: 30-day GN-BSI-related mortality 16.8% (19/113); all-cause mortality 26.5% (30/113). Transplant-type signal: carbapenem-resistant GN-BSI more frequent in liver vs. kidney recipients.	Most common organisms: *E. coli* 38.9% (46/118), *K. pneumoniae* 34.7% (41/118), *A. baumannii* 10.2% (12/118). Resistance markers: ESBL production (44.9%), CR-R (35.6%). CR-R GB-BSI was higher in LT vs. KT(69.2% vs. 17.6%, *P* < 0.001). Primary infection sites: UTI 54.0%, surgical site 31.9%.
[31]	Zebian G. *et al.*	France	Annals of Intensive Care; 2024	Retrospective single-centre observational cohort (Jan–Dec 2020)	Cohort size: 1313 ICU patients; 271 (20.6%) immunocompromised. 28-day cumulative incidence of ICU-acquired bacterial BSI:- Immunocompromised: 27/271 (10.0%); - Nonimmunocompromised: 115/1042 (11.0%) Association with mortality: ICU-acquired BSI was not significantly associated with 28-day ICU mortality.	Gram-negative bacilli (~64%) of BSI, mainly *K. pneumoniae* and *Enterobacter* spp. MDR: 47/142 (33.1%) episodes due to MDR bacteria; similar between immunocompromised and nonimmunocompromised groups.
[32]	Kamaladasa D. *et al.*	Australia	TID; 2025	Single-centre retrospective cohort study	Burden: 31 episodes of CPE isolation from 28 liver transplant recipients: 9 infections + 22 new colonizations. Infection sites (*n* = 9): urinary tract 3, bloodstream 3, wound 2, 1 GI. Incidence rate comparison: CPE infection rate higher in LT vs. general hospital population: 1.92 vs. 0.30 per 10 000 occupied bed days (*P* = 0.04).	Species: *Klebsiella pneumoniae* most common (12/31, 39%), then *E. coli* (13%), K. oxytoca (13%), *Citrobacter freundii* (10%), *Enterobacter hormachei* (10%), etc. Carbapenemase genes: NDM 42%, OXA/SME 29%, IMP 19%, plus NDM+OXA 4%
[33]	Liu L. *et al.*	China	Microbiology Spectrum, 2025	Retrospective single-centre observational cohort of consecutive adult liver transplant recipients (2010–2023)	Infection burden: 776 LT recipients; 156 (20.1%) developed post-LT infection; 207 pathogens from 180 infection sites. Sites reported include pulmonary infection, peritonitis, cholangitis, abdominal abscess, pleural effusion, catheter-related sepsis, UTI. MDR burden (overall, not BSI-specific): “ > 45%” of bacterial infections caused by drug-resistant pathogens; “ > 30%” of GNB carbapenem-resistant.	Overall pathogens: GNB 90 isolates (43.5%), GPB 82 (39.6%), fungi 35 (16.9%). Main GNB species: *A. baumannii* (33), *K. pneumoniae* (24), *E. coli* (14), *E. cloacae* (5), *P. aeruginosa* (6), S. maltophilia (4). Drug-resistant GNB categories: CRE 8 (7 *K. pneumoniae*, 1 *E. coli*), ESBL 7 (5 *E. coli*, 2 *E. cloacae*), CRAB 14, CRPA 2.
[9]	Boutzoukas A. E. *et al.*	US	AJT; 2025	Matched study within two prospective, multicentre observational cohorts	121 SOT vs. 242 non-SOT BSI infection 17% (21/121)	CRE: *K. pneumoniae* (70%), *E. cloacae* (12–13%), *E. coli* (9–10%)
[34]	Genovese C. *et al.*	Italy	TID; 2025	Multicentre retrospective observational cohort (registry-based; 2018–2024)	Population: 2210 SOT recipients (≥48h ICU stay) admitted to ICU during the same hospitalization as transplant. ICU-acquired infections (ICU-HAI): 154/2210 (6.97%) developed 193 ICU-HAIs; median onset 6 days from ICU admission and 6 days from transplant. BSI burden: 56/193 (29%) of ICU-HAIs were bloodstream infections (BSI). Mortality impact: intra-ICU mortality 22.4% with ICU-HAI vs. 2.4% without; intra-hospital mortality 7.0% vs. 1.7%. MDRO subgroup outcomes: intra-ICU mortality 23.5% (MDRO) vs. 17.0% (non-MDRO) and longer ICU/hospital LOS (trend).	Microbiology available for 87/154 (56.5%) infected patients; GNB predominated overall (≈60–75% by year). MDRO prevalence: 34/87 (39%). CR-GNB prevalence 25.3% (22/87). Most common MDROs reported: carbapenem-resistant *Klebsiella* spp. (and, among GPB, VRE E. faecium).
[35]	Tanriverdi E. S. *et al.*	Turkey	Diagnostic Microbiology & Infectious Disease; 2026	Retrospective single-centre microbiology-based cohort (blood-culture CRKP isolates in LT, 2016–2022)	Incidence of CRKP bacteraemia in LT recipients (study period): 127/2100 (6.05%) 30-day mortality: 51.2% (65/127). 90-day mortality: 64.6%	Carbapenemase genes (*n* = 127), OXA-48 59.1%, OXA-48 + NDM 17.3%, NDM 10.2%, KPC 3.9%, IMP 3.1%, VIM 1.6% CZA-S overall: 77.9% susceptible, 22.0% resistant By gene: OXA-48 98.6% susceptible; OXA-48+NDM 54.5%; NDM 23.0%; KPC 100%; IMP 25%; VIM 0%

aHR, adjusted hazard ratio; aOR, adjusted odds ratio; BSI, bloodstream infection; carba-R, carbapenem-resistant/resistance; CAZ-AVI, ceftazidime-avibactam; CPE, carbapenemase-producing Enterobacterales; CP-Kp, carbapenemase-producing *Klebsiella pneumoniae*; CR, carbapenem-resistant; CRAB, carbapenem-resistant *Acinetobacter baumannii*; CRE, carbapenem-resistant Enterobacterales; CRGNB/CR-GNB, carbapenem-resistant Gram-negative bacteria; CRKP, carbapenem-resistant *Klebsiella pneumoniae*; CRPA, carbapenem-resistant *Pseudomonas aeruginosa*; CR-R, carbapenem-resistant; CZA-S, ceftazidime–avibactam susceptible; EB, Enterobacterales; ESBL, extended-spectrum β-lactamase; ESBL-E, ESBL-producing Enterobacterales; ESBL-EB, ESBL-producing Enterobacterales; ESBL-GNBSI, ESBL Gram-negative bloodstream infection; FQ-R, fluoroquinolone-resistant/resistance; GI, gastrointestinal; GN, Gram-negative; GNB, Gram-negative bacteraemia; GNBSI/GN-BSI, Gram-negative bloodstream infection; IAI, intra-abdominal infection; ICU, intensive care unit; ICU-HAI, ICU-acquired healthcare-associated infections; IMP, IMP-type metallo-β-lactamase (“imipenemase”); KPC, *Klebsiella pneumoniae* carbapenemase; KT, kidney transplant; LT, liver transplant; MDR, multidrug-resistant; MDRO, multidrug-resistant organism; N/A, not available/not applicable; NDM/NDM-1, New Delhi metallo-β-lactamase; OXA-48, OXA-48-like carbapenemase; PTZ-R, piperacillin–tazobactam-resistant/resistance; SOT, solid organ transplant; TMP-SMX, trimethoprim–sulfamethoxazole; Tx, transplantation; UTI, urinary tract infection; VIM/VIM-1, Verona integron-encoded metallo-β-lactamase (variant VIM-1); XDR, extensively drug-resistant.

### Patients with haematological malignancies

Patients with HM are particularly susceptible to bloodstream infections due to prolonged and profound neutropenia, severe mucositis, frequent antibiotic exposures, and gastrointestinal colonization by MDR organisms [43–45]. The incidence (Table 2) [2,5,45–54] of Gram-negative BSI is high, with cumulative rates of 17.3% at 30 days after allogeneic HSCT and 9% at 20 days after autologous HSCT [2]; overall, Gram-negative bacteria account for 42–63% of all BSI episodes in this population [5,50]. The predominant pathogens are *E. coli* (27–52%), *K. pneumoniae* (6–28%), and *P. aeruginosa* (9–16%) [2,5,50]. Multidrug resistance rates are alarmingly high, with MDR Gram-negative bacteria responsible for 30–67% of Gram-negative BSI episodes [4^▪▪^,^50^,^50^]. ESCR-E rates range from 17 to 66%, while CRE rates from 13% to 39%, with particularly high rates observed in *K. pneumoniae* (27–57%) and *P. aeruginosa* (27–56%) [49,50,55^▪▪^]. In contrast to SOT recipients, mortality associated with GNBSI in haematological patients is substantially higher, with overall 30-day mortality rates of 16–21% and significantly increased mortality in MDR infections (27–34% vs. 7–8% in non-MDR; OR 5.02) [47,48]. The highest mortality rates are observed with carbapenem-resistant *A. baumannii* (56%) and metallo-β-lactamase-producing *K. pneumoniae* (47–55%) [48]. Importantly, the risk of recurrence and poor outcomes is strongly influenced by the timing of BSI relative to the disease course and treatment phase. BSI occurring ≥30 days after HSCT is independently associated with markedly increased mortality (odds ratio 11.21), reflecting the impact of disease status, ongoing immunosuppression, and cumulative treatment-related toxicity on infection outcomes [1].

**Table 2:** Epidemiology and aetiology of MDRGNBSI in haematological patients

Ref	First author	Country	Journal and year of publication	Study type	Epidemiology	Aetiology
[45]	Cattaneo C. *et al.*	Italy (multicentre, SEIFEM Group)	Ann Hematol; 2018	Multicentre prospective observational study (6-month period; 2226 admissions)	MDR rectal colonization detected in 144/2226 admissions (6.5%). Among colonized patients, 37/144 (25.7%) developed ≥1 BSI. MDR-related BSI occurred in 23/144 (16% overall; 62.2% of BSIs). MDR-related BSI occurred mainly during neutropenia (87%). 30-day mortality from colonization: 6.9%; mortality strongly associated with carbapenem-resistant (CR) MDR-related BSI.	ESBL-producing Enterobacteriaceae (64/144): ESBL-Escherichia coli (*n* = 50), ESBL-*Klebsiella pneumoniae* (*n* = 6), ESBL-Enterobacter spp. (*n* = 5). Carbapenem-resistant (CR) Gram-negative bacteria (85/144): CR-*Klebsiella pneumoniae* (*n* = 37), CR-Escherichia coli (*n* = 20), CR-Enterobacter spp. (*n* = 5), CR-*Citrobacter freundii* (*n* = 3), CR-*Morganella morganii* (*n* = 2), CR-Proteus mirabilis (*n* = 3), MDR-Acinetobacter spp. (*n* = 9), MDR-*Pseudomonas aeruginosa* (*n* = 10). Gram-positive: Vancomycin-resistant *Enterococcus* spp. (VRE) (*n* = 9). MDR-related BSIs were mainly due to ESBL-*E. coli*, ESBL-*K. pneumoniae*, CR-*K. pneumoniae*, CR-Enterobacter aerogenes, CR-*P. aeruginosa*, CR-Acinetobacter spp., and VRE.
[46]	Torres I. *et al.*	Spain	BMC Infectious Disease; 2022	Single-centre retrospective observational study (2015–2019); 361 admissions in 250 adult haematological patients	BSI occurred in 102/361 admissions (28%). MDRB-BSI significantly more frequent in colonized vs. noncolonized patients (16/47 vs. 9/55; *P* = 0.04). Colonization independently associated with MDRB-BSI (HR 3.70; 95% CI 1.38–9.90; *P* = 0.009).	Gram-negative MDR organisms (89.9% of colonizing isolates): ESBL-producing Escherichia coli (*n* = 36), plasmidic AmpC *E. coli* (*n* = 5), ESBL-*Klebsiella pneumoniae* (*n* = 6), plasmidic AmpC *K. pneumoniae* (*n* = 2), ESBL-Enterobacter cloacae (*n* = 1), VIM-type carbapenemase-producing *K. pneumoniae* (*n* = 1); MDR-*Pseudomonas aeruginosa* (*n* = 37), including VIM-type carbapenemase producers (*n* = 16); MDR-Stenotrophomonas maltophilia (*n* = 27); MDR-Acinetobacter spp. (*n* = 3).
[47]	Trecarichi M. *et al.*	Italy (15 centres, HEMABIS–SEIFEM registry)	International Journal of Antimicrobial Agents, 2023	Multicentre prospective cohort study (January 2016–December 2018) including 811 consecutive GNB episodes	811 GNB BSI episodes with 834 Gram-negative isolates. Most common pathogens: *E. coli* (52.7%), *K. pneumoniae* (19.2%), *P. aeruginosa* (14.6%). MDR isolates accounted for 256/834 (30.7%). Carbapenem-resistant Enterobacterales: 13.9%; third-generation cephalosporin-resistant Enterobacterales: 37.4%. 30-day mortality: 16.3%; significantly higher in MDR GNB BSI (34.4% vs. 7.9%, *P* < 0.001). MDR GNB BSI independently associated with mortality (OR 5.02).	Enterobacterales: *Escherichia coli* (440 isolates; 17.1% MDR), *Klebsiella pneumoniae* (160; 63.1% MDR), *Enterobacter cloacae* (31; 12.9% MDR). Nonfermenters: *Pseudomonas aeruginosa* (122; 36.9% MDR), *Acinetobacter baumannii* (14; 64.3% MDR, all XDR), *Stenotrophomonas maltophilia* (14; 100% MDR). Notable resistance patterns: carbapenem resistance in 34.4% of *P. aeruginosa* and 64.3% of *A. baumannii*; meropenem susceptibility in *K. pneumoniae* 48.7%.
[5]	Averbuch D. *et al.*	Multinational (65 HSCT centres, 25 countries across Europe, Asia, Australia)	Clinical Infectious Diseases, 2017	Intercontinental prospective multicentre observational study (Feb 2014–May 2015) including HSCT recipients within 6 months posttransplant	655 GNR-BSI episodes in 591 HSCT patients; 704 Gram-negative isolates. 50% resistant to fluoroquinolones and noncarbapenem anti-Pseudomonas β-lactams; 18.5% carbapenem-resistant; 35.2% MDR. Resistance rates significantly higher in allogeneic vs. autologous HSCT (MDR 43.7% vs. 20.2%, *P* < 0.001). 7-day mortality 6.5%, significantly higher in carbapenem-resistant (18% vs. 4%) and MDR infections (11% vs. 4%).	Enterobacteriaceae (73%): *Escherichia coli* (43%), *Klebsiella pneumoniae* (18%), *Enterobacter* spp., *Citrobacter* spp., *Serratia* spp., *Proteus* spp., *Raoultella* spp. Nonfermenters (24%): *Pseudomonas aeruginosa* (14%), *Acinetobacter* spp., *Stenotrophomonas maltophilia* (all considered MDR). Carbapenem resistance mainly observed in *K. pneumoniae* and nonfermenters; non-*Klebsiella* Enterobacteriaceae rarely C-R.
[48]	Falcone M. *et al.*	Italy (19 hospitals, ALARICO Network)	JAC Antimicrobial Resistance, 2025	Prospective multicentre observational study (June 2018–January 2020) including hospitalized cancer patients with GNB bloodstream infection	347 cancer patients with GNB BSI; 66.9% (232/347) caused by MDR-GNB. Overall 30-day mortality: 20.5%. Mortality significantly higher in MDR-GNB vs. susceptible GNB (27.2% vs. 7%, *P* < 0.001). Highest mortality in CRAB (56.3%) and MBL-producing *K. pneumoniae* (55.6%). MDR-GNB independently associated with 30-day mortality. Attributable mortality to MDR-GNB: 43% (ESBL 33%, KPC 32%, MBL 47%, CR-NFGNB 73%).	Susceptible GNB (33.1%): *Pseudomonas aeruginosa*, *Escherichia coli*, *Klebsiella pneumoniae*, *Proteus mirabilis*, *Enterobacter* spp., *Acinetobacter* spp., *Citrobacter freundii*, *Serratia marcescens*, others. MDR-GNB (66.9%): ESBL-producing Enterobacterales (34.6%), KPC-producing *Klebsiella pneumoniae* (19%), MBL-producing *K. pneumoniae* (5.2%), carbapenem-resistant *Pseudomonas aeruginosa* (3.5%), carbapenem-resistant *Acinetobacter baumannii* (4.6%).
[49]	Kayaaslan B. *et al.*	Turkey (Ankara Bilkent City Hospital)	Tropical Medicine and Health, 2025	Retrospective observational single-centre study (June 2019–September 2023) including adult hematologic and oncologic cancer patients with Gram-negative BSI	435 patients with 569 Gram-negative BSI episodes. Median age 56 years; 60.7% male. 51.5% hematologic and 48.5% solid malignancies. MDR rate 47.7%; ESBL production 26.7%; carbapenem resistance 13%. Most frequent pathogens: *E. coli* (40.6%), *Klebsiella* spp. (28.8%), *Pseudomonas* spp. (9.1%). Catheter-related BSI 11.1% overall (20.5% in hematologic vs. 1.1% in solid tumours, *P* < 0.001).	Enterobacterales: *Escherichia coli* (ESBL 40.3%, carbapenem resistance 9.1%, MDR 49.8%); *Klebsiella* spp. (ESBL 28.7%, carbapenem resistance 27.4%, MDR 56.1%); *Enterobacter* spp. (MDR 37.9%, carbapenem resistance 13.8%). Nonfermenters: *Pseudomonas* spp. (carbapenem resistance 27.1%, MDR 23.1%); *Acinetobacter baumannii* (carbapenem resistance 52.2%, MDR 56.5%); *Stenotrophomonas maltophilia* (intrinsic resistance; TMP-SMX susceptibility 57.1%).
[47]	.	Italy (9 tertiary/university haematology centres; HeMABIS–SEIFEM registry)	Clinical Microbiology and Infection, 2015	Multicentre prospective cohort study (January 2009–December 2012) including adult patients with hematologic malignancies and bacterial bloodstream infection	575 BSI episodes (668 isolates; 14.4% polymicrobial). GN-BSI accounted for 52.8% of isolates. Among monomicrobial BSI, 21-day mortality significantly higher for Gram-negative vs. Gram-positive infections (16.9% vs. 5.6%, *P* < 0.001). Resistance to third-generation cephalosporins in Enterobacteriaceae: 36.9%. Carbapenem-resistant *K. pneumoniae*: 34.9%. MDR *P. aeruginosa*: 69.7%. Inappropriate empirical therapy associated with higher mortality (22.5% vs. 10.1%, *P* < 0.001).	Gram-negative bacteria (52.8%): *Escherichia coli* (27.9%), *Pseudomonas aeruginosa* (9.9%), *Klebsiella pneumoniae* (6.4%), *Enterobacter cloacae* (3.4%), *Acinetobacter baumannii*, *Stenotrophomonas maltophilia*. High resistance patterns: third-generation cephalosporin-resistant Enterobacteriaceae (36.9%); carbapenem-resistant *K. pneumoniae* (34.9%); MDR *P. aeruginosa* (69.7%).
[51]	Royo-Cebrecos. C. *et al.*	Multinational (34 centres, 12 countries; IRONIC study group)	Pathogens, 2022	Multicentre retrospective cohort study (January 2006–May 2018) including adult neutropenic cancer patients with *Pseudomonas aeruginosa* bloodstream infection	1217 PA BSI episodes: 917 in HM and 300 in ST. HM patients more frequently had profound neutropenia (67% vs. 44.6%, *P* < 0.001). ST patients more often presented with septic shock (45.8% vs. 30%, *P* < 0.001) and had higher 30-day mortality (49% vs. 37.3%, *P* < 0.001). Inadequate empirical antibiotic therapy more frequent in HM (20.1% vs. 12%, *P* < 0.001). Independent predictors of 30-day mortality included high-risk MASCC score, septic shock, inappropriate empirical therapy, MDRPA and pneumonia (HM), persistent BSI (ST).	*Pseudomonas aeruginosa* (study focused on PA BSI). MDRPA defined as nonsusceptible to ≥1 agent in ≥3 antimicrobial categories; XDRPA defined as nonsusceptible to all but ≤2 categories. MDRPA more frequent in HM (23.2%) than ST (7.7%).
[52]	Amanati A. *et al.*	Iran (Shiraz, single referral oncology hospital)	BMC Infectious Diseases, 2021	Retrospective single-centre study (July 2015–August 2019) including adult cancer patients with confirmed BSI	414 positive blood cultures (from 2393 tested). Gram-negative bacteria accounted for 63.3% (262/414). Overall mortality 21.5% (7-day mortality 10%; 30-day mortality 3.4%). Among Enterobacterales and nonfermenters (*n* = 257), 39.3% were carbapenem-resistant. ESBL producers 64.1% among Enterobacteriaceae. MDR Gram-negative BSI 49.3%. Carbapenem-resistant GN increased annually (*P* < 0.001). Resistant gram-positive BSI associated with higher mortality (35.4%, *P* = 0.001).	Gram-negative (63.3%): *Escherichia coli* (47%), *Pseudomonas* spp. (31%), *Klebsiella pneumoniae* (14.5%), *Acinetobacter* spp., *Enterobacter* spp. ESBL rates: *E. coli* 66.7%, *K. pneumoniae* 60.7%, *Pseudomonas* spp. 96.2%, *Acinetobacter* spp. 100%. Carbapenem resistance: *Acinetobacter* spp. 77.8%, *Pseudomonas* spp. 70.7%, *Enterobacter* spp. 33.3%, *E. coli* 24.4%, *K. pneumoniae* 13.2%. Overall MDR GN 49.3%.
[53]	Rosa, R. G. *et al.*	Brazil (Hospital de Clínicas de Porto Alegre)	Antimicrobial Agents and Chemotherapy, 2014	Prospective single-centre cohort study (October 2009–August 2011) including adult cancer patients with FN	307 FN episodes (169 patients); 115 episodes (37.4%) had BSI. Overall 28-day mortality 9.4% (29 deaths). BSI and relapsing disease independently associated with mortality. TTA independently predicted 28-day mortality (HR 1.18 per hour delay; 95% CI 1.10–1.26). TTA ≤30 min associated with significantly lower mortality compared to 31–60 min (3.0% vs. 18.1%, *P* = 0.0002).	Bloodstream isolates (37.4% of FN episodes): *Escherichia coli* (41.7%), *Klebsiella pneumoniae* (11.3%), *Pseudomonas aeruginosa* (9.5%), *Serratia* spp., *Enterobacter* spp.
[2]	Girmenia C. *et al.*	Italy (54 transplant centres; GITMO–AMCLI network)	Clinical Infectious Diseases, 2017	Prospective multicentre observational survey (January–December 2014) including 1118 allo-HSCT and 1625 auto-HSCT recipients	Cumulative incidence of preengraftment GNB: 17.3% at 30 days after allo-HSCT and 9% at 20 days after auto-HSCT. Overall 149 GNB in allo-HSCT (12.5%) and 151 in auto-HSCT (9%). Preengraftment GNB independently associated with increased 4-month mortality in both allo-HSCT (HR 2.13; 95% CI 1.45–3.13; *P* < 0.001) and auto-HSCT (HR 2.43; 95% CI 1.22–4.84; *P* = 0.01).	Most frequent pathogens: *Escherichia coli* (allo 51.7%; auto 60.9%), *Klebsiella pneumoniae* (allo 18.8%; auto 15.2%), *Pseudomonas aeruginosa* (allo 14.1%; auto 8.6%). Resistance patterns (allo-HSCT): cephalosporin-nonsusceptible *E. coli* ≈39%; carbapenem-nonsusceptible *K. pneumoniae* ≈57%; MDR *P. aeruginosa* ≈38%.
[54]	Puerta-Alcalde P. *et al.*	Spain	Transplantation and Cellular Therapy, 2021	Prospective observational cohort study (2008–2017) including HSCT recipients with BSI	402 BSI episodes in 293 HSCT recipients (75.4% allogeneic; 32.3% autologous; 19.3% second HSCT). Median time from HSCT to BSI: 62 days (IQR 9–182). Overall 30-day mortality: 19.2%. Inappropriate empiric antibiotic therapy in 26.6%. Independent risk factors for 30-day mortality: BSI ≥30 days after HSCT (OR 11.21), shock (OR 7.10), BSI caused by MDR *Pseudomonas aeruginosa* (OR 4.45), and inappropriate empiric therapy for Gram-negative bacilli or *Candida* spp. (OR 3.73).	Gram-negative bacilli (42%): *Pseudomonas aeruginosa* (15.9%), *Escherichia coli* (13.4%; 31.5% ESBL), *Klebsiella pneumoniae* (5.5%). Overall MDR Gram-negative bacilli: 11.9% (48 episodes), including 31 MDR *P. aeruginosa* (48.4% of all pseudomonal BSI) and 17 ESBL *E. coli*. Carbapenem-resistant *P. aeruginosa* accounted for 56.3% of pseudomonal isolates.

allo-HSCT, allogeneic hematopoietic stem cell transplantation; AmpC, AmpC beta-lactamase; auto-HSCT, autologous hematopoietic stem cell transplantation; BSI, bloodstream infection; CR, carbapenem-resistant; CRAB, carbapenem-resistant *Acinetobacter baumannii*; CRE, carbapenem-resistant Enterobacterales; CR-GNB, carbapenem-resistant Gram-negative bacilli; CR-NFGNB, carbapenem-resistant nonfermenting Gram-negative bacilli; CRPA, carbapenem-resistant *Pseudomonas aeruginosa*; ESBL, extended-spectrum beta-lactamase; ESBL-EC, extended-spectrum beta-lactamase–producing *Escherichia coli*; FN, febrile neutropenia; GNB, Gram-negative bacilli; GNR, Gram-negative rods; HM, hematologic malignancies; HSCT, hematopoietic stem cell transplantation; KPC, *Klebsiella pneumoniae* carbapenemase; MBL, metallo-beta-lactamase; MDR, multidrug-resistant; MDRB, multidrug-resistant bacteria; MDRB-BSI, bloodstream infection caused by multidrug-resistant bacteria; MDRPA, multidrug-resistant *Pseudomonas aeruginosa*; MRSA, methicillin-resistant *Staphylococcus aureus*; PA, *Pseudomonas aeruginosa*; SEIFEM, Italian Hematologic Infections Surveillance Group; ST, solid tumours; TMP-SMX, trimethoprim-sulfamethoxazole; TTA, time to antibiotic administration; VIM, Verona integron-encoded metallo-beta-lactamase; VRE, vancomycin-resistant *Enterococcus*; XDR, extensively drug-resistant.

## EMPIRICAL THERAPY

### Solid organ transplant recipients

As in the general population, empirical therapy for suspected BSI in SOT recipients is based on clinical severity (e.g. presence of septic shock, qSOFA and SOFAscore calculation [56]), local epidemiology and individual risk factors for MDR. In centres with a high prevalence of ESCR-E, carbapenems remain the treatment of choice [3]. However, in specific circumstances carbapenem-sparing options could be considered, mainly for clinically stable patients with BSI from urinary sources in hospitals where activity of old β-lactam/β-lactamase inhibitor as piperacillin/tazobactam is still preserved [22]. In addition, new carbapenem-sparing alternatives (e.g. cefepime-enmetazobactam) are currently available and could be employed in these clinical scenarios [57,58].

In several hospitals, particularly in Europe [60], rectal screening for MDR-GN carriage is done [59]. Initially adopted for infection control purposes [60], systematic screening for MDRO-carriage status is now increasingly used to tailor surgical prophylaxis [61,62] and/or to guide empirical therapy in case of infection symptoms onset [11]. Nevertheless, two critical aspects warrant consideration. First, screening protocols must be aligned with local epidemiology and laboratory capacity, ensuring the selection of appropriate and affordable detection methods (e.g. culture-based and/or molecular plus antibiogram including new drugs). Moreover, MDR-GN colonization, in particular CRE, should be assessed both pretransplantation and periodically postsurgery, as the early posttransplant period carries the highest risk for new colonization and hospital-acquired infections [11]. Second, since rectal colonization alone has a positive predictive value (PPV) of approximately 50% [64], empirical MDR-GN coverage in clinically stable carriers with suspected BSI should take into account additional risk factors, as multisite colonization and/or complicated posttransplant course [11].

### Patients with haematological malignancies

The approach to empirical therapy in HM patients with neutropenic fever is well established in recent guidelines [55^▪▪^]. Clinical severity and local epidemiology, along with a history of prior infection or colonization, are the primary drivers for the use of broad-spectrum agents, in particular carbapenems. As previously highlighted, sparing antibiotics with activity against anaerobic pathogens may be beneficial in this setting. However, the available evidence is still limited and potentially confounded by disease severity, indication bias, and heterogeneity in patient management [14–16]. A colonization driven approach in HM settings has been recognized as a useful strategy for the empirical therapy of febrile neutropenia with GNBSI, given the evidence of better outcomes in colonized patients receiving appropriate initial therapy, mainly with the use of novel Bl/BLIs [8,55^▪▪^,^65^]. Similar to SOT, this approach requires a predefined screening protocol addressing target pathogens, screening sites, culture vs. molecular upfront tests and frequency of screening. In this regard, data remain limited: two surveys, one European and one from Belgium, reported that while rectal colonization screening for ESBL and CPE is standard in most centres, approaches to *P. aeruginosa* and MRSA screening remain heterogeneous [66,67].

Finally, in patients at high risk for CPE or difficult to treat resistant *P. aeruginosa* infections, the European Conference on Infections in Leukaemia (ECIL) has endorsed the empirical use of new BL/BLI combinations [55^▪▪^].

## APPROACH TO QUASI-TARGETED THERAPY

Currently, the antimicrobial management of severe infections and infections caused by MDR pathogens is closely linked to the availability and implementation of advanced diagnostic techniques. These tools offer rapid turnaround time, high sensitivity, specificity, and negative predictive value. However, their widespread use in immunocompromised host patients is not supported by robust, population-specific validation studies. There still remain concerns about off-target pathogens, and uncertainty regarding optimal integration into diagnostic algorithms and antimicrobial stewardship programs [68,69].

**FIGURE 1:**
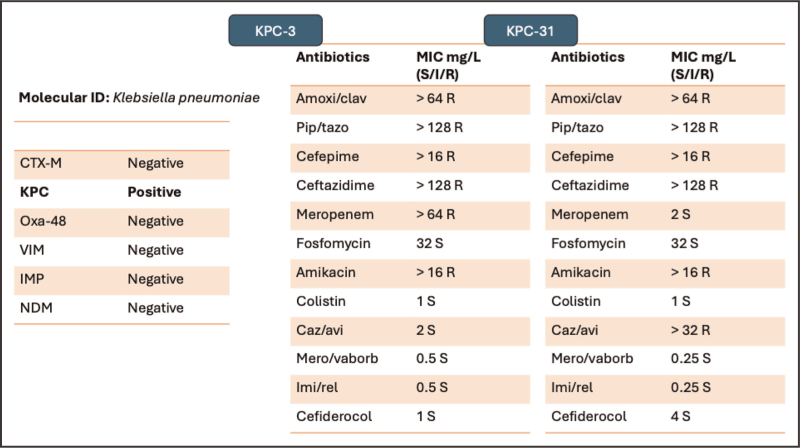
Example of *Klebsiella pneumoniae* producing KPC carbapenemase, illustrating that the molecular resistance mechanism does not always translate into a uniform phenotypic antibiogram, with in this case variable susceptibility to ceftazidime–avibactam due to the production of a KPC variant (KPC-31).

Indeed, a consensus document addressing the use of rapid diagnostic tools in SOT recipients [68] recommended that, in patients with Gram-negative BSI, preliminary molecular test results should be used to guide escalation of anti-Gram-negative coverage, but not de-escalation. This caution is warranted because antimicrobial resistance in Gram-negative bacteria can be mediated by multiple mechanisms that are not consistently detected by currently available molecular panels (Fig. 1). Rapid phenotypic tests for the prompt detection of MDR in severe patients with GN-BSI are currently available, but their impact on clinical outcome is still under investigation [70]. Finally, limited accessibility and high costs of these technologies represent further barriers to their widespread implementation.

Taking these considerations into account, an artificial intelligence tool has been recently developed to predict resistance to the main anti-Gram-negative antibiotic classes, using a large cohort of patients with Gram-negative BSI [71]. This tool was designed to be applied upon pathogen identification by MALDI-TOF mass spectrometry and was built using readily obtainable clinical variables as site of BSI acquisition, colonization status, and principal comorbidities. Other studies have described machine learning–based risk stratification tools capable of combining prior MDR colonization data in patients with febrile neutropenia, thereby supporting personalized empirical therapy in this population [72,73].

## APPROACH TO TARGETED THERAPY

Targeted therapy for MDR-GNBSI in immunocompromised patients should adhere to current guideline recommendations, with novel BL/BLI combinations and cefiderocol primarily reserved for carbapenem-resistant strains [55^▪▪^,^74^,^75^]. The longstanding debate regarding combination therapy for *P. aeruginosa* infections remains unresolved; however, available evidence favours its use in patients with haematological malignancies who present with critical illness and/or pneumonia as the source of bloodstream infection [55^▪▪^].

**FIGURE 2:**
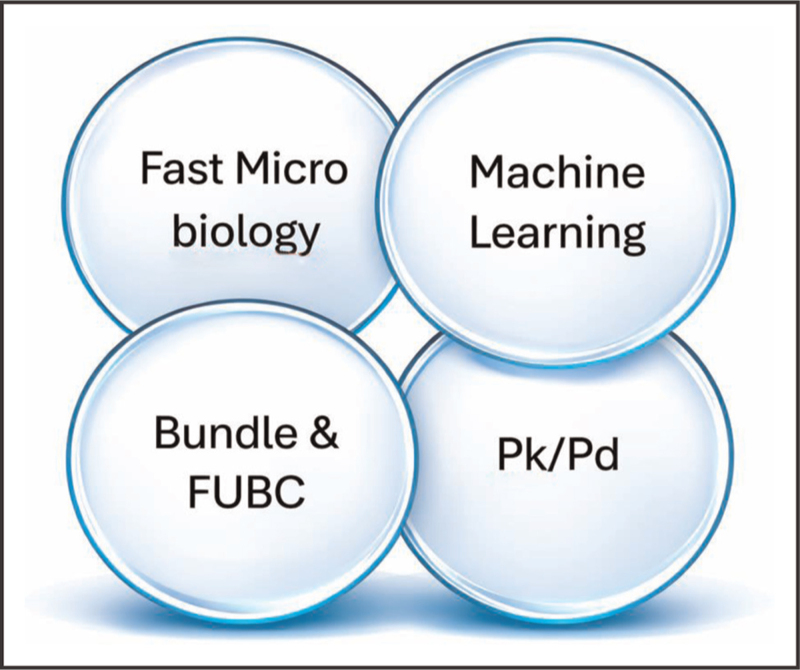
Overview of innovative strategies in the management of MDR Gram-negative bloodstream infections in immunocompromised patients.

Regarding administration schedules, continuous or extended infusion of novel β-lactams has been associated with higher rates of PK/PD target attainment and favourable outcomes in liver transplant recipients, but only in uncontrolled observational studies [76] (Fig. 2). Data in HM patients are more controversial, with one randomized controlled trial demonstrating no benefit from continuous infusion of β-lactams [77], while another supported this strategy [78].

Moreover, a critical issue with novel BL/BLI combinations is the imbalanced pharmacokinetic profile between the two components, with the inhibitor often exhibiting suboptimal exposure relative to the β-lactam, in particular for avibactam. This disproportion may facilitate the selection of resistant mutants, as inadequate β-lactamase inhibitor concentrations fail to fully protect the partner β-lactam from enzymatic degradation [79]. Therapeutic drug monitoring allows real-time dose adjustments to restore an appropriate balance between the two components, thereby optimizing target attainment and minimizing the risk of resistance emergence [80].

Transition to oral therapy and shortening of treatment duration are key components of antimicrobial stewardship programs [81^▪▪^] (Fig. 2). In SOT recipients, oral therapy for GNBSI has demonstrated comparable efficacy to intravenous treatment; however, it should be noted that oral options for MDRGN remain limited [82]. Consequently, the optimal strategy in these cases is to minimize treatment duration whenever clinically appropriate. Importantly, the presence of MDR alone does not constitute an indication for prolonged therapy, and preliminary data support shorter treatment courses for both CRE [83] and *P. aeruginosa* bloodstream infections [84], even in ICHs.

A debated component of MDRO GNBSI management is the routine use of follow-up blood cultures (FUBCs) (Fig. 2). In several cohorts, FUBCs have shown a relatively low microbiological yield and may increase the risk of contaminant (false-positive) results, potentially leading to prolonged antibiotic courses and longer hospital stays [39^▪^]. Conversely, observational studies have also reported an independent association between FUBC performance and reduced mortality, likely reflecting closer clinical monitoring and more timely source-control interventions, both key to microbiological clearance and relapse prevention that, as mentioned above, seems to be particularly frequent in SOT recipients compared to other patient groups with GNBSI [6,85]. Importantly, the available evidence is almost entirely observational; therefore, residual confounding and biases (including selection and immortal-time bias) may have influenced these findings. Randomized data are awaited to clarify the net clinical benefit of systematic FUBCs [86].

Finally, a structured Gram-negative BSI bundle was proposed [87] and recently assessed in clinical practice [88]. No significant reduction in mortality was observed, whereas the intervention was associated with decreased new MDR-GN colonization and subsequent infections, suggesting a measurable impact on MDRO containment and prevention despite the absence of a detectable survival effect.

## CONCLUSION

The management of multidrug-resistant GNBSI in immunocompromised patients remains a significant clinical challenge, with rising rates of ESCR-E, CRE, and difficult to treat resistant *P. aeruginosa* contributing to substantial morbidity and mortality.

While the availability of novel antimicrobial agents has broadened the spectrum of effective targeted regimens, optimal management continues to depend on early administration of in-vitro active therapy, timely and adequate source control, and structured monitoring to ensure microbiological clearance and reduce the risk of relapse. The integration of rapid diagnostic platforms, PK/PD-optimized dosing strategies, oral step-down therapy and bundle-based approaches has the potential to improve both clinical outcomes and preserve gut microbiome; however, these interventions require robust prospective validation in immunocompromised populations [63].

## Acknowledgements

*None.*

### Financial support and sponsorship

*None.*

### Conflicts of interest

*There are no conflict of interest.*

## REFERENCES AND RECOMMENDED READING

Papers of particular interest, published within the annual period of review, have been highlighted as:

▪ of special interest
▪▪ of outstanding interest
